# Letter from the Editor in Chief

**DOI:** 10.19102/icrm.2022.130308

**Published:** 2022-03-15

**Authors:** Moussa Mansour


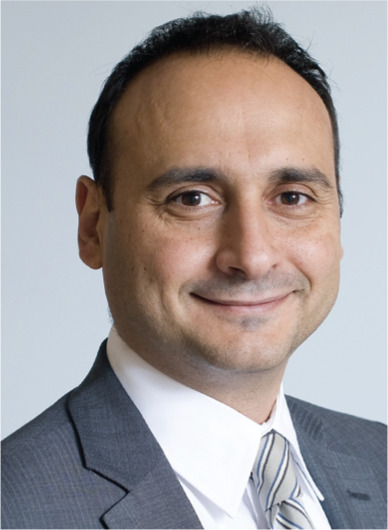


Dear readers,

This issue of *The Journal of Innovations in Cardiac Rhythm Management* contains an important article by Mehta et al. titled “Utility of Ischemia Testing Prior to Ablation for Sustained Monomorphic Ventricular Tachycardia.” In this retrospective study, the authors compared clinical outcomes between patients who underwent ischemia workup and revascularization prior to ablation for monomorphic ventricular tachycardia (VT) and those who did not.^1^ They found that an ischemia workup, although frequently completed, infrequently led to revascularization and did not have a clinical benefit.

Although this study is retrospective and included only a small number of patients, it is important because it highlights a common and clinically relevant topic. Although monomorphic VT has been well established as a result of pre-existing scar, ischemia evaluation continues to be performed in a large number of cases prior to VT ablation. There are a few possible reasons for this practice, and some are discussed in the abovementioned article. Some prior reports and studies reported a reduction or elimination of VT after revascularization. Another reason could be a concern for hemodynamic instability during ablation in the presence of flow-limiting coronary artery disease. However, it is rare that such practice results in revascularization and improvement of the clinical outcome. A pre-ablation ischemia workup also carries some disadvantages, perhaps the most significant of which is a delay in the procedure.

This study is hypothesis-generating and hopefully will lead to a prospective clinical trial. Until then, I believe that it might impact clinical practice and would lead some clinicians, including myself, to pause a bit before performing an ischemia workup prior to ablation and instead perhaps adopt an individualized approach where such testing is performing only in situations of a clear threat of hemodynamic instability during the procedure secondary to ischemia.

I hope that you enjoy reading this issue of *The Journal of Innovations in Cardiac Rhythm Management*.

Sincerely,


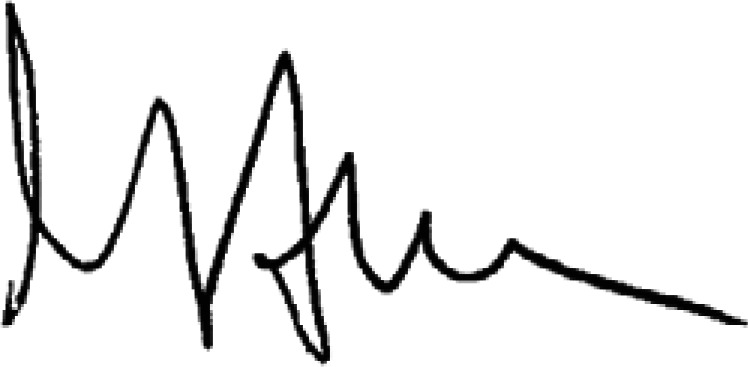


Moussa Mansour, md, fhrs, facc

Editor in Chief

*The Journal of Innovations in Cardiac Rhythm Management*

MMansour@InnovationsInCRM.com

Director, Atrial Fibrillation Program

Jeremy Ruskin and Dan Starks Endowed Chair in Cardiology

Massachusetts General Hospital

Boston, MA 02114
